# Modeling and Measurement of Correlation between Blood and Interstitial Glucose Changes

**DOI:** 10.1155/2016/4596316

**Published:** 2016-04-27

**Authors:** Ting Shi, Dachao Li, Guoqing Li, Yiming Zhang, Kexin Xu, Luo Lu

**Affiliations:** ^1^College of Electronic Information and Control Engineering, Beijing University of Technology, Beijing 100124, China; ^2^State Key Laboratory of Precision Measuring Technology and Instruments, Tianjin University, Tianjin 300072, China; ^3^Department of Medicine, University of California School of Medicine, Torrance, CA 90502, USA

## Abstract

One of the most effective methods for continuous blood glucose monitoring is to continuously measure glucose in the interstitial fluid (ISF). However, multiple physiological factors can modulate glucose concentrations and affect the lag phase between blood and ISF glucose changes. This study aims to develop a compensatory tool for measuring the delay in ISF glucose variations in reference to blood glucose changes. A theoretical model was developed based on biophysics and physiology of glucose transport in the microcirculation system. Blood and interstitial fluid glucose changes were measured in mice and rats by fluorescent and isotope methods, respectively. Computer simulation mimicked curves were fitted with data resulting from fluorescent measurements of mice and isotope measurements of rats, indicating that there were lag times for ISF glucose changes. It also showed that there was a required diffusion distance for glucose to travel from center of capillaries to interstitial space in both mouse and rat models. We conclude that it is feasible with the developed model to continuously monitor dynamic changes of blood glucose concentration through measuring glucose changes in ISF with high accuracy, which requires correct parameters for determining and compensating for the delay time of glucose changes in ISF.

## 1. Introduction

Assessment of glucose concentration changes in the blood by a continuous blood glucose monitoring is important for the diagnosis and treatment of diabetes. Several methods that have been developed for continuous blood glucose monitoring by other labs include using a near infrared spectroscopy, microdialysis, enzyme electrode sensors, ultrafiltration, and open-flow microperfusion [1–5]. Up to date, there is a lack of the reliable method that can be applied to measure the blood glucose concentration through continuously monitoring changes in the interstitial space fluid (ISF). It will be a significant development if ISF glucose readout can accurately reflect the blood glucose changes. Recent studies that focus on improving the precision and accuracy of ISF glucose measurements demonstrate an accurate mathematical model to calculate delay of the changes in glucose in ISF by comparison with the changes of glucose in blood. They found that it is rather challenging to convert ISF glucose changes into the blood glucose concentrations because of the problem that applied mathematical models may not accurately reflect the time delay of ISF rising following the changes in blood glucose concentration [6]. In other studies, ISF glucose changes are discussed by using a human subcutaneous tissue model into a mass transfer model formula in reference to glucose changes in the capillaries [7]. Combined mass transfer and black box-like models are developed with noninvasive methods and relied on input and output glucose values for detecting blood glucose concentrations, respectively [8–10]. The disadvantage of these studies is that they rely on a few unknown parameters for explaining such a complex system [11]. In a more advanced study model, the time delay measured is for 10 min following a glucose concentration gradient in the default setting of calculating blood glucose concentrations according to values of the ISF glucose concentration [12]. However, all of these studies could not provide a precise description for glucose exchange dynamics between blood and interstitial compartments, because there is a little or lack of consideration concerning some physiological parameters in the microcirculation system, including permeability coefficient, blood pressure drops in the capillary, and glucose concentration gradients that directly affect transport and diffusion processes.

Later, there was a more comprehensive model developed by using a glucose sensor that is implanted in interstitial space and contains glucose oxidase to generate electric currents. By using this method, ISF glucose changes can be continuously monitored in reference to blood glucose concentrations [13]. However, results obtained with this method may not accurately reflect the true ISF glucose value because the implanted electrode sensor is easily clotted, resulting in potential drifts. The question remains whether ISF glucose concentration changes can be accurately measured consistently with the blood glucose changes. The present study aims to establish a mathematical model based on physiology of microcirculation. We combine the model with real-time measurements of the glucose dynamics in blood and ISF compartments, which can accurately predict blood glucose concentrations from continuous measurements of ISF glucose contents. We included in the study the kinetic analysis of glucose movement from capillaries to surrounding tissues by taking into consideration concentration gradient differences and blood pressure-driven force involving capillary walls, ISF, and diffusion process within the interstitial space. In addition, a novel isotope-labeling method was also used to be compared with the fluorescent indicator method. A time delay for glucose concentration changes from the blood circulation to ISF was calculated by a theoretical model fitted with our experimental data obtained from both fluorescent sensor method in mice and isotope-tracer method in rats.

## 2. Materials and Methods

### 2.1. Glucose Diffusion Equations

Arterioles carry the glucose in the blood circulation to the capillaries allowing glucose to diffuse across the capillary wall into interstitial space. After exchange, glucose flows out of the capillaries and into the venule and then the veins [14]. These processes can be explained by Fick's permeability equation and Johnson and Wilson equation, Starling-Landis law, and Fick's second law, respectively.* First*, diffusion is the most important way that moves glucose across capillary walls. According to Fick's permeability equation [15], the diffusion flux of glucose molecules between blood and tissue was given as follows: *J*_*s*_ = *P* · (*C*_blood_ − *C*_ISF_), where *J*_*s*_ is the glucose flux of concentration gradient-driven transport from blood to tissue compartment, *P* is the permeability coefficient, *C*_blood_ is the blood glucose concentration, and *C*_ISF_ is the ISF glucose concentration.* Second*, as a result of glucose diffusion and changes in transport through capillary walls from blood to tissue, the glucose concentrations in venous blood can be very different from those in arterial blood. The glucose extraction (*E*) from blood perfusing to tissue was calculated from the arterial (*C*_*a*_) and venous (*C*_*v*_) glucose concentration as *E* = (*C*_*a*_ − *C*_*v*_)/*C*_*a*_, where *E* is the glucose extraction coefficient, *C*_*a*_ is the arterial glucose concentration, and *C*_*v*_ is the venous glucose concentration. The total glucose mass lost or gained by the blood was calculated by the equation of amount of glucose lost/gained per min = *E* × *F* × *C*_*a*_, where *F* is the blood flow.* Finally*, the total mass of glucose lost/gained per min across the diffusion area (*A*) was revised as

(1) CISF-Fickt=Cbloodt−1−e−PA/F×F×CbloodtPA.

### 2.2. Glucose Concentration and Blood Pressure Gradient-Driven Transport

The interrelationship between hydrostatic pressure and oncotic forces within the capillaries and tissue was described by Starling law [16]. In our calculation, we assumed that the distance from the arteriole to the venule is a unit of 1.0; when *x* = 0, the capillary pressure (*P*_*c*_) is equal to the arteriolar pressure (*P*_*a*_). When *x* = 1.0, the capillary pressure is equal to the venular pressure (*P*_*v*_). The loss flux from plasma is the same as the gain flux to the tissue, defined as *J*_*V*-gain_. Similarly, the fluid gain to plasma is the same as the fluid loss from tissue, assuming *J*_*V*-loss_. However, interstitial hydrostatic and oncotic pressure are too small to be included. The amount of flux gain to, or loss from, the tissue can be deduced from the integral area as the following:

(2) The  fluid  gain  to  tissue  is  JV-gain=Kf∫PaσπcPc−PaPv−Pa·dPc,The  fluid  loss  from  tissue  is  JV-loss=Kf∫σπcPvPv−PcPv−Pa·dPc.

The fluid gained from the capillary would mix with the fluid in the tissue. Therefore, the whole fluid in tissue included some from capillary and the rest from tissue. The net amount of glucose in ISF close to the capillary wall can therefore be calculated as

(3) CISF-Starlingt=JV-gainJV-gain+JV-loss·Cbloodt+JV-lossJV-gain+JV-loss·CISFt.

### 2.3. Glucose Diffusion in Tissue

After glucose passes through the capillary wall into tissues, it diffuses throughout the tissue in multiple directions. The random order of glucose diffusion in the tissue is related to both the diffusion time and distance. The glucose transport within tissue was calculated according to Fick's 2nd law [15]. We assumed the tissue as a semi-infinite bar with a constant source (blood) of glucose molecules diffusing from a finite to an infinite side. At the very beginning of the diffusion process (*t* = 0), the ISF glucose concentrations are the same throughout in tissue without a gradient, defined as the initial condition *C*{*x*, *t* = 0} = *C*_ISF0_. The ISF glucose concentrations close to the capillary wall are determined only by the amounts of blood glucose molecule crossing the capillary wall and are unaffected by glucose from other areas, described as *C*{*x* = 0, *t*} = *C*_blood-ISF_(*t*). Taking both conditions together, we derived the following equation, where *D* is the diffusion coefficient and *x* is diffusion distance:

(4) CISFx,t=Cblood-ISFt−Cblood-ISFt−CISF0·erf⁡x2D·t,

where erf is the error function; the error function equation was written as

(5) $$\begin{array}{c} \mathrm{erf}\left(\;z\;\right)=\frac{2}{\sqrt{\pi }}\overset{z}{\underset{0}{\int }}\exp\left\{\;-u^{2}\;\right\}\cdot du. \end{array}$$

This integral equation was resolved by fitting experimental data numerically with a computer, and so erf results found in error function table were used to solve the diffusion equation (4) where necessary.

### 2.4. Glucose Detection with Fluorescent Indicator in Mice

In the previous studies, we have developed a fluorescent indicator for measurements of glucose changes in mice. In brief, glucose concentrations in blood and ISF were continuously monitored by an embedded glucose sensitive fluorescent gel in test tubes and in the abdominal areas in mice, respectively. This method is based on a competitive reaction among borate polymer, poly(acrylamide-ran-3-acrylamidophenylboronic acid) (PAA-ran-PAAPBA) [17], alizarin, and glucose [18]. Alizarin, a nonfluorescent substance, reacts with the borate polymer to produce the fluorescent alizarin-borate polymer (ABP). When glucose specifically binds to the borate polymer in ABP, a competitive reaction occurs, causing the fluorescent ABP to decompose into nonfluorescent alizarin. Consequently, changes in fluorescence intensity can be quantified and calibrated to determine glucose concentrations. Fluorescent ABP was prepared using 30 mg of high molecular weight carboxylated poly(vinylchloride), 60 *μ*L of bisebacate (2-ethylhexyl), 4 mg of tridodecylmethylammonium chloride, and 1 mg of alizarin from Fluka (St. Louis, MO, USA) and 3 mg of borate polymer (PAA-ran-PAAPBA) obtained from the University of South Carolina [19]. These materials were placed into a glass vial and dissolved in 500 *μ*L of tetrahydrofuran (THF, ≥99% purity; Sigma, St. Louis, MO, USA). The mixture was vortexed to form a gel structure after a 4 h dry process [20]. Fluorescence intensity of the embedded gel was first recorded* in vivo* for the background fluorescent intensity in ISF by an imaging system (IVIS-100 Bioluminescence Machine; Caliper Company), and then a 50% glucose solution (400 *μ*L/100 g body weight) was injected into the peritoneum, and the fluorescence intensity was recorded by IVIS every 10 min with excitation at 460 nm and emission at 570 nm [21]. At the same time points, obtained venous blood samples with 2 mL/tube were transferred to a 96-well microplate containing ABP and photographed.

### 2.5. Blood and ISF Glucose Measurement by Using ^3^H Isotope in Rats

Change of the glucose concentrations in the blood circulation and ISF was measured through an isotope-labeling method. In the studies, 12–16-week-old female Wistar rats were used with similar size and body weight (200–300 g). The rats were fasted overnight before the experiment and maintained by feeding normal water during fasting. Before experiments, the basal glucose levels were measured by sampling blood sugar through the caudal vein. Animals were anesthetized with isoflurane and injected with 25% glucose solution that contained ^3^H-labeled glucose (3 *μ*Ci/20 g body weight) for the intravenous glucose tolerance tests (GTT). The isotope ^3^H was not enriched during the experiments. After injection of the glucose solution for 5 min, blood samples were immediately collected from one of the rats for measuring blood glucose concentration (*C*_blood_) using a glucose meter and ^3^H isotope content in blood sample (*T*_blood_) was also measured by using a liquid scintillation counter.

## 3. Results

### 3.1. Glucose Compartmental Microcirculation Model

**Figure 1:**
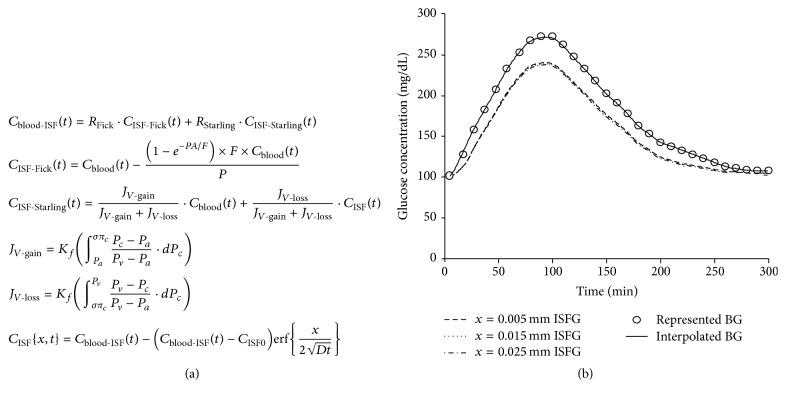
Theoretical model and simulation of correlation of blood and ISF glucose changes. (a) Mathematical equations derived from Starling equation and Fick's laws for study of the relationship between blood and interstitial fluid (ISF) glucose changes. (b) Computer simulation generated time courses of glucose concentration changes in blood and ISF. The solid line represents blood glucose changes. The other dashed lines represent the ISF glucose changes after transcapillary transport through the capillary walls. Glucose concentration changes were simulated by the theoretical model with various diffusion distances (*x*) of 5, 15, and 25 *μ*m. BG and ISFG represent blood and ISF glucose, respectively.

The whole process of glucose exchange between capillaries and surrounding tissues includes transcapillary transport of glucose to the tissue and diffusion of glucose in the tissue. A model was developed to describe this process with derived equations from Starling-Landis law and Fick's second law (Figure 1(a)). An assumption was made based on unified distribution of microcirculation in the skin, muscle, and surrounding tissues. Thus, the sum of *R*_Fick_ and *R*_Starling_ is 1. Therefore, the model can be applied disregarding where the glucose sensor was placed. In addition, detection tissues include the skin. Since the skin is unlikely to have significant glucose uptake, the glucose uptake in skin tissue can be ignored [8]. Based on microcirculation physiology, computer simulation modeling curves were presented using the derived equations (Figure 1(b)). A given group of the blood glucose concentrations with a linear interpolation was used as the input parameters in the simulation in reference to ISF glucose concentration. As shown in the modeling curves, blood and ISF glucose concentrations have the same variation tendency. When blood glucose levels increased, ISF glucose levels followed and vice versa. However, blood and ISF glucose concentrations were not equivalent at each time point, suggesting that there were delayed changes in ISF when glucose levels rapidly changed in the blood.

### 3.2. Measurement of Blood and ISF Glucose Changes with Fluorescent Indicator in Mice

**Figure 2:**
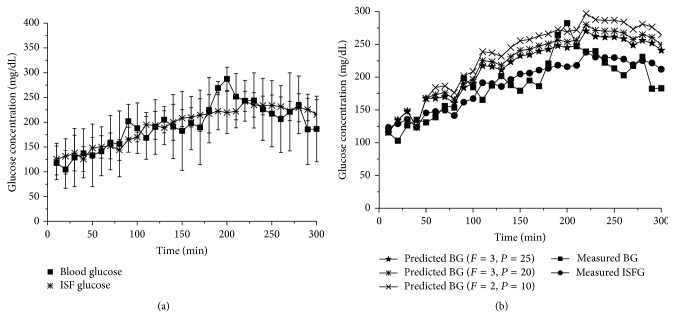
Measurements of blood and ISF glucose changes in mice. (a) Time course of blood and ISF glucose concentration changes in mice. (b) Comparisons of glucose changes measured in blood (BG) and interstitial fluid (ISFG) of mice with the time courses generated by the theoretical simulations. For the* in vivo* studies, six experimental mice (three male and three female) with similar size and weight were used. The mice were fasted for one night before the experiment. *F* is the blood flow and *P* is the glucose extraction from blood related to glucose membrane permeability ensued in the theoretical model.

The correlation of blood and ISF glucose concentration changes was measured by detecting intensity of fluorescent alizarin-borate polymer (ABP) that was embedded under the skin in abdominal region of mice. Glucose specifically binds to the borate polymer in ABP resulting in fluorescent ABP to decompose into nonfluorescent alizarin and changed fluorescence intensity can be quantified to determine the concentrations of the added glucose molecules. At the same time points, venous blood samples were obtained and blood glucose concentrations were measured in a 96-well microplate containing ABP. A time course of fluorescent intensity changes that were calibrated to reflect glucose concentrations was plotted to demonstrate ISF and reference blood glucose concentrations (Figure 2(a)). Changes of blood and ISF glucose concentrations measured by fluorescent detection method verified the mathematical model presented in Figure 1. All values obtained from measurement of ISF glucose concentrations in mice were plotted in the mathematical simulation model to yield the referenced blood and ISF glucose concentrations with previous fixed physiological parameters (Figure 2(b)). The maximum and average relative error (abs(measured − predicted)/measured) were 38.16% and 16.42% (*F* = 3, *P* = 25), 43.01% and 19.12% (*F* = 3, *P* = 20), and 51.72% and 24.29% (*F* = 2, *P* = 10), respectively. Results of measured time courses of blood and ISF glucose concentrations with fluorescent method were matched with predicted blood and ISF glucose concentration changes simulated by the mathematical simulation model, indicating that the model simulated blood and ISF glucose changes may be used for prediction of the correlation of blood and ISF glucose levels following a physiologically related time course.

### 3.3. Measurement of Blood and ISF Glucose Changes with Isotope-Labeled Glucose in Rats

**Figure 3:**
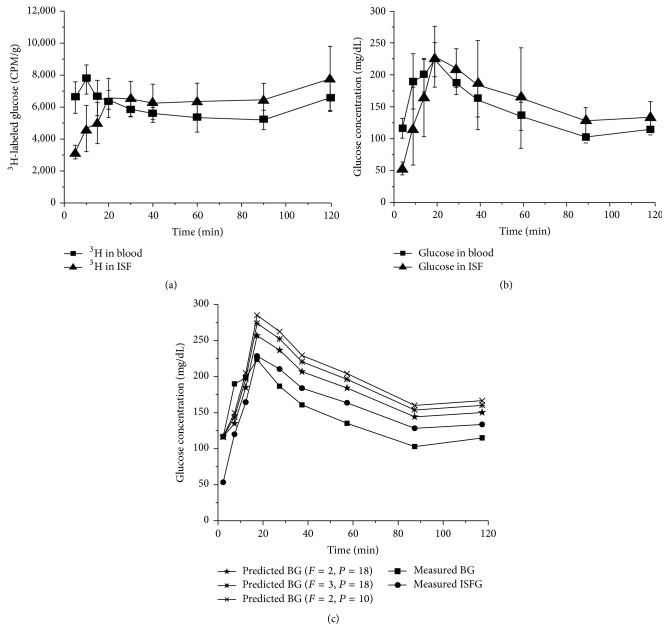
Measurements of blood and ISF glucose changes with ^3^H-labeled glucose in rat. (a) Time course of ^3^H-labeled glucose activity measured in blood and ISF of rats. (b) Time course of glucose concentration changes in blood and ISF of rats. (c) Comparisons of glucose changes measured in blood (BG) and interstitial fluid (ISFG) of rats with the time courses generated by the theoretical simulations. *F* is the blood flow and *P* is the glucose extraction from blood related to glucose membrane permeability ensued in the theoretical model. The changes of ^3^H isotope levels in blood and tissue can reflect the changes of glucose levels in these two compartments after isotope activity was converted with the equation of *T*_blood_/(*T*_ISF_) = *C*_blood_/*C*_ISF_. *T*_blood_ and *T*_ISF_ are ^3^H isotope activities in blood and ISF, respectively. *C*_blood_ and *C*_ISF_ represent blood glucose and ISF glucose concentrations, respectively. For the* in vivo* studies, 5 experimental rats with similar size and weight were used.

Blood and ISF glucose concentrations were measured by using an isotope-tracer method with ^3^H-labeled glucose in rats. Concentrations of ^3^H-labeled glucose in blood were increased in a very fast time course after intravenous injection. However, changes of ^3^H isotope in ISF were significantly delayed about 10 min to reach the peak point when compared with blood isotope counts and then decreased following a similar time course as the blood ^3^H isotope changes (Figure 3(a)). After calculation of ^3^H-labeled glucose in reference to total glucose, glucose concentrations were converted and plotted to show that changes of the glucose concentration in blood and ISF had a similar time course as seen in initially measured ^3^H isotope time course except that the concentration of blood glucose reached the highest point at the same time as the peak time of ISF glucose concentration (Figure 3(b)). Results of the time course in ^3^H-labeled glucose experiments in rats were plotted to be compared with computer-simulated curves with the mathematical model. The relationship between the time course of glucose concentration changes in ISF or blood measured by ^3^H-labeled glucose and the time course simulated with the mathematical model was fairly consistent, where the maximum and average relative errors, (abs(measured − predicted)/measured), were 40.28% and 23.73% (*F* = 2, *P* = 18), 49.56% and 28.24% (*F* = 3, *P* = 18), and 55.61% and 31.92% (*F* = 2, *P* = 10), respectively (Figure 3(c)). Predicted changes of blood and ISF glucose concentrations by computer simulation demonstrated a rapid increase after injection of glucose solution to reach the peak concentration at 20 min, and then glucose concentrations decreased to a stationary phase in 80 min. The exact patterns found in changes of ISF and blood glucose concentrations in experimental rats were observed. Thus, the mathematical model and computer simulation data can be validated by a combination of theoretical and experimental approaches in rats* in vivo* to dynamically predict the changes of glucose concentrations in blood related to those in ISF.

### 3.4. Prediction of Delayed ISF Glucose Changes

**Figure 4:**
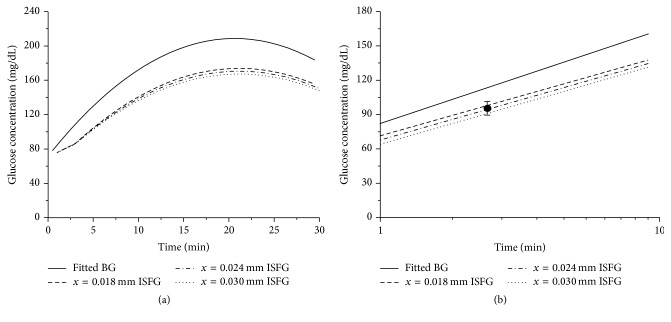
Diffusion and delay of glucose from blood to ISF in mice. (a) Prediction of ISF glucose changes following different diffusion distances (*x*). (b) Projection of measured ISF glucose changes to the predicted time course of ISF glucose changes in semilog curves. A better fitting time point was found in a diffusion distance of 24 *μ*m in mice, indicating a time delay for glucose changes in ISF compared with blood glucose changes.

**Figure 5:**
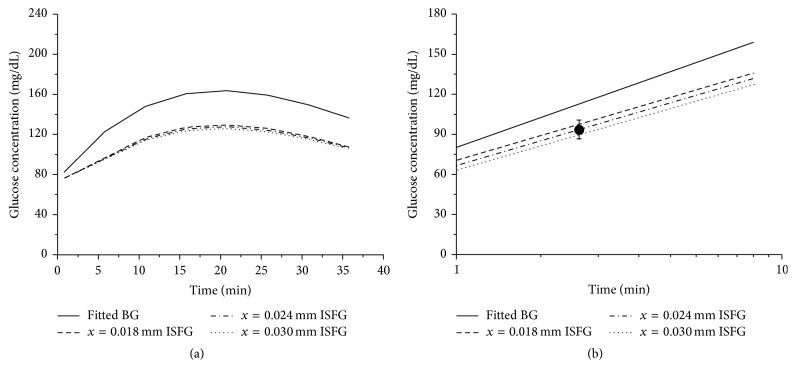
Diffusion and delay of glucose from blood to ISF in rats. (a) Prediction of ISF glucose changes following different diffusion distances (*x*). (b) Projection of measured ISF glucose changes in rats to the predicted time course of ISF glucose changes in semilog curves. The curve with a diffusion distance of 24 *μ*m was fitted better by measured value of ISF glucose changes in rats, indicating a time delay for glucose changes in ISF compared with blood glucose changes.

In the mouse model, a time course reflecting the blood glucose and ISF concentrations after glucose injection was plotted with the theoretical model with predicted diffusion distances (*x* = 18, 24, and 30 *μ*m). Experimental data obtained in mice using the fluorescent gel sensor method were projected to the theoretical curves with an estimated value of 2.0 min at a diffusion distance of 24 *μ*m from the center of a capillary to interstitial space in both lineal and semilog plots (Figures 4(a) and 4(b)). A time course of the blood glucose and ISF level changes after injection of glucose in rats were also plotted by using the isotopic tracer ^3^H-labeled glucose with predicted diffusion distances (*x* = 18, 24, and 30 *μ*m). Experimental data obtained from measurements of glucose concentration changes in rats were projected to the theoretical curves with an estimated value of 2.4 min at a diffusion distance of 24 *μ*m from the center of a capillary to interstitial space in both lineal and semilog plots (Figures 5(a) and 5(b)). These results for the first time demonstrated the time delays for glucose rising in ISF in reference to the glucose changes in the blood circulation in mice and in rats.

## 4. Discussion

The objective of this study is to establish an accurate theoretical model based on biophysical and physiological properties of the microcirculation to calculate the correlation and difference in timing between the blood and ISF glucose concentration changes. Several factors cause concentration differences and a time-lag between blood and ISF glucose levels, including different distributions of glucose between blood vessels and subcutaneous tissue, the permeability of glucose, blood flow, and changes in the release of pancreatic hormones such as insulin and glucagon [22, 23]. A delayed relationship therefore exists between blood and ISF glucose concentrations, especially when glucose levels are rapidly changing. A theoretical model is developed in this study to simulate the correlation and time course of glucose changes in the blood circulation and in ISF according to biophysics and physiology of microcirculation. Glucose molecules moving through blood vessel into ISF can be described by Starling equation and then diffusing in ISF follows the principles of Fick's laws. The mathematical model used in this study combines Starling equation and Fick's laws to accurately reflect the entire process of glucose molecule transport in the microcirculation system. Glucose concentration changes in the blood and ISF were mimicked and time course curves were generated by a computer simulation (Figure 1).

In the early publications, many of the results are confounded by calibration errors and the fact that early generation CGM sensors impose a long filtering lag time [13]. Recently, new studies conducted by this group recalibrated the lag time and evaluated facts that affect this assessment [24]. In the present study, two approaches have been developed using fluorescent and isotope methods to measure the relationship between blood and ISF glucose changes in mice and rats, respectively. Increased glucose concentration deassociated alizarin from alizarin-borate polymer (ABP) to bleach the fluorescent intensity of ABP gel that was embedded under the skin in abdominal regions [18]. Fluorescent intensity changes of ABP gel were recorded by a fluorescent camera in a photo documentary system specifically designed for mice. Measurements of glucose levels in rats were performed by using ^3^H-labeled glucose, since rats provide more skin-covered areas than mice for sampling in the time course studies. In time course studies, blood and interstitial glucose changes demonstrated similar trends in increase and decline phases of the glucose levels measured from mice and rats. We note that the predicted values in results are actually higher than the values obtained from experimental measured values of blood and ISF glucose concentrations, especially appearing in the decline phase of glucose changes. This is probably because the model used does not reflect the endogenous regulatory decrease in glucose concentrations, which is controlled by insulin and the nervous system in living animals. To answer this very challenging question of regulatory mechanisms, the applied models in future studies should include the endogenous regulatory mechanism to further improve the accuracy of the measurements. By inclusions of microcirculation parameters according to mouse and rat anatomic characteristics, the theoretical modeling curves generated by computer simulation demonstrated synchronized time courses for blood and ISF glucose changes compared with the time courses biologically measured from mouse and rat blood and ISF glucose changes.

In the study, we have developed a theoretical and mathematical model based on Starling equation and Fick's laws to accurately describe the glucose transport in the microcirculation. Compared with other methods used in measurements of blood and ISF, we were able to incorporate parameters measured from different animal models and different methods that can read out and track glucose movements in the blood and ISF. One of the important parameters that are closely related to geographic and biological structures in the microcirculation is the diffusion distance “*x*” that describes how far glucose molecules diffuse from the center of a capillary to reach an average point of interstitial space in ISF. To validate the theoretical model, time delays of glucose changes in ISF compared with the blood were calculated from fluorescent method in mice and ^3^H-labeled isotope method in rats and plotted into computer-simulated curves (Figures 4 and 5). Interestingly, the best fitting time points for both fluorescent and isotope methods from respective mice and rats were falling to curves with the same “*x*” value of 24 *μ*m (Figures 4(b) and 5(b)). As demonstrated in the study, potential delay times for the glucose molecules from the center of the capillary blood to ISF were 2.0 ± 0.5 min and 2.4 ± 0.8 min for mice and rats, respectively. The difference of the delay times between mice and rats may result from the methods of measurements and differences in characteristics of vascular permeability. However, the difference presented in this study is rather small in terms of the allowed range or the data validation. In conclusion, the kinetics of glucose movement from capillaries to tissues have been considered and described, which include concentration gradient-driven transport and pressure gradient-driven transport between capillaries and tissue and diffusion process within tissue. Meanwhile, physiological parameters, such as glucose permeability and blood flow, have been introduced into the model to reflect the delay in transport. Both fluorescent and isotope methods were developed to determine the correlation between glucose concentration changes in the blood circulation and ISF and calculate the time delay relationship in mice and rats. In addition, a theoretical model has been verified by simulating glucose dynamics based on model formulae, physiological parameters, and fitting modeling curves with measured glucose concentration changes following time courses. Most of the parameters used in the study models should be compatible with human subjects because of the similarities in the microcirculation system between human and rat/mouse. However, some parameters may require to be modified for human use since there are differences in thickness and hairy sinks between human and rat/mouse. In future, we will proceed with testing the model by adjusting the parameters in different conditions such as diabetes from other species.
