# Corrigendum to “Modeling and Measurement of Correlation between Blood and Interstitial Glucose Changes”

**DOI:** 10.1155/2017/3164027

**Published:** 2017-08-20

**Authors:** Ting Shi, Dachao Li, Guoqing Li, Yiming Zhang, Kexin Xu, Luo Lu

**Affiliations:** ^1^School of Precision Instrument and Opto-Electronics Engineering, Tianjin University, Tianjin 300072, China; ^2^College of Electronic Information and Control Engineering, Beijing University of Technology, Beijing 100124, China; ^3^Department of Medicine, Division of Molecular Medicine, Harbor-UCLA Medical Center, David Geffen School of Medicine at the University of California, Los Angeles, CA 90095, USA; ^4^Los Angeles Biomedical Research Institute at Harbor-UCLA Medical Center, Torrance, CA 90502, USA

In the article titled “Modeling and Measurement of Correlation between Blood and Interstitial Glucose Changes” [1], there were errors in the authors' affiliations. The correct affiliation list is shown above.
